# Antioxidant Activities, Phenolic Compounds, and Sensory Acceptability of Kombucha-Fermented Beverages from Bamboo Leaf and Mulberry Leaf

**DOI:** 10.3390/antiox12081573

**Published:** 2023-08-06

**Authors:** Ruo-Gu Xiong, Si-Xia Wu, Jin Cheng, Adila Saimaiti, Qing Liu, Ao Shang, Dan-Dan Zhou, Si-Yu Huang, Ren-You Gan, Hua-Bin Li

**Affiliations:** 1 Guangdong Provincial Key Laboratory of Food, Nutrition and Health, Department of Nutrition, School of Public Health, Sun Yat-sen University, Guangzhou 510080, China; xiongrg@mail2.sysu.edu.cn (R.-G.X.); wusx6@mail2.sysu.edu.cn (S.-X.W.); chengj225@mail2.sysu.edu.cn (J.C.); saimaiti@mail2.sysu.edu.cn (A.S.); zhoudd6@mail2.sysu.edu.cn (D.-D.Z.); huangsy9@mail2.sysu.edu.cn (S.-Y.H.); 2 School of Chinese Medicine, Li Ka Shing Faculty of Medicine, The University of Hong Kong, Hong Kong 999077, China; liuqing2@connect.hku.hk (Q.L.); shangao@connect.hku.hk (A.S.); 3 Singapore Institute of Food and Biotechnology Innovation (SIFBI), Agency for Science, Technology and Research (A*STAR), 31 Biopolis Way, Singapore 138669, Singapore

**Keywords:** kombucha, mulberry leaf, bamboo leaf, fermentation, antioxidant activities

## Abstract

Kombucha is traditional drink made from the fermentation of a black tea infusion, and is believed to offer a variety of health benefits. Recently, exploring kombucha made from alternative substrates has become a research hotspot. In this paper, two novel kombucha beverages were produced with bamboo leaf or mulberry leaf for the first time. Moreover, the effects of fermentation with leaf residues (infusion plus residues) or without leaf residues (only infusion) on the antioxidant properties of kombucha were compared. The ferric-reducing antioxidant power assay, Trolox equivalent antioxidant capacity assay, Folin–Ciocalteu method, and high-performance liquid chromatography were utilized to measure the antioxidant capacities, total phenolic contents, as well as some compound concentrations of the kombucha. The results showed that two types of kombucha had high antioxidant capacities. Moreover, kombucha fermented with bamboo leaf residues (infusion plus residues) significantly enhanced its antioxidant capabilities (maximum increase 83.6%), total phenolic content (maximum increase 99.2%), concentrations of some compounds (luteolin-6-C-glucoside and isovitexin), and sensory acceptability, compared to that without residues (only infusion). In addition, fermentation with leaf residues had no significant effect on mulberry leaf kombucha. Overall, the bamboo leaf was more suitable for making kombucha with residues, while the mulberry leaf kombucha was suitable for fermentation with or without residues.

## 1. Introduction

Kombucha is a popular drink that is traditionally made by fermenting a mixture of black tea infusion and sugar with the symbiotic culture of bacteria and yeast (SCOBY) [1]. The fermentation process converts the sweetened tea infusion into an acidic taste and slightly fizzy drink. Moreover, several studies showed that kombucha contained a variety of bioactive components, and possessed abundant bioactivities, such as antioxidant activity, anti-inflammation, immune regulation, anti-diabetes, anti-obesity, hepatoprotection, blood pressure regulation, and anticancer effects [2,3,4,5,6]. Several tea and non-tea plants have been tested as alternative substrates for kombucha production, such as green tea, oolong tea, vegetables, fruits, and medicinal herbs, each with its unique chemical components that may influence the bioactivities of the final kombucha product [7,8].

The mulberry leaf contains many bioactive compounds, such as chlorogenic acid, rutin, and astragalin [9,10]. It is widely utilized in traditional medicines and functional foods due to its numerous health benefits, including antioxidant, anti-inflammatory, anti-diabetes, cardiovascular protective, and anticancer activities, and so on [11,12]. On the other hand, the bamboo leaf has been widely used in functional foods and traditional medicines, and provides multiple health benefits, including antioxidant properties, cardiovascular protection, anti-obesity, anti-diabetes, etc. [13,14]. Several studies suggested that the high content of phenolic compounds in the bamboo leaf were significant contributors to its health benefits [15,16]. There is no existing literature that has reported on the kombucha beverages made by the bamboo leaf or mulberry leaf. In addition, kombucha fermentation with tea residues, such as black tea, green tea, and sweet tea, could enhance the antioxidant properties as well as polyphenol contents of beverages according to our previous studies [8,17]. In this study, we investigated the antioxidant activities, polyphenol contents, as well as sensory acceptability of kombucha beverages made from mulberry leaf and bamboo leaf, and compared the impact of fermentation with or without leaf residues on these indicators. This study could be helpful for the valorization of the cheap mulberry and bamboo leaves.

## 2. Materials and Methods

### 2.1. Materials and Reagents

Mulberry leaf was bought from Anhui Tongjuntang Biotechnology Co., Ltd. (Bozhou, China). Fresh bamboo leaf was obtained in Guangzhou city (Guangzhou, China) and then was dried in air. The kombucha starter culture was purchased from Shandong Ruyun Edible Fungus Planting Co., Ltd. (Liaocheng, China).

Folin–Ciocalteu’s phenol reagent, gallic acid, 2,20-azinobis (3-ethylbenothiazoline-6-sulfonic acid) diammonium salt (ABTS), 2,4,6-tri(2-pyridyl)-S-triazine (TPTZ), and 6-hydroxy-2,5,7,8-tetramethylchromane-2-carboxylic acid (Trolox) were acquired from Sigma-Aldrich (St. Louis, MO, USA). Acetic acid, ethanol, hydrochloric acid, iron(II) sulfate heptahydrate, iron(III) chloride hexahydrate, potassium persulfate, and sodium acetate were procured from Tianjin Chemical Factory (Tianjin, China). Sodium carbonate was bought from Shanghai Yuanye Biological Technology Co., Ltd. (Shanghai, China). Sucrose, methanol, and formic acid were purchased from Macklin Chemical Factory (Shanghai, China). The standard chemicals, involving astragalin, chlorogenic acid, caffeine, ellagic acid, epicatechin, gallic acid, kaempferol, quercetin, quercitrin, isovitexin, luteolin-6-C-glucoside, and rutin, were obtained from Derick Biotechnology Co., Ltd. (Chengdu, China).

### 2.2. Activation of Kombucha Starter Culture

This procedure was performed following the manufacturer’s instructions. Briefly, 5 g teabag of black tea, 100 g sucrose and 1 L boiled water were added into a flask and mixed thoroughly. The teabag was removed after a 5 min soaking period and the mixture was cooled to room temperature (25 °C). The kombucha fungus, fermented broth, and cellulosic layer were then added to the mixture. The mixture was then placed into a clean and dark environment for 14 days at room temperature for subsequent inoculation.

### 2.3. Kombucha Preparation Based on Bamboo Leaf and Mulberry Leaf

The kombucha beverages were divided into 4 groups: (a) kombucha based on bamboo leaf with residues; (b) kombucha based on bamboo leaf without residues; (c) kombucha based on mulberry leaf with residues; (d) kombucha based on mulberry leaf without residues. In each glass flask, 200 mL of distilled water and 20 g of sucrose were added and heated in a boiling water bath. Then, 2 g of mulberry leaf or bamboo leaf was put into the flasks and kept for 5 min. After cooling to room temperature, the infusion was obtained by filtering the mixture through a sieve for fermentation without residues. In the case of fermentation with residues, filtration was skipped. Then, 20 mL of activated kombucha starter culture was added to each flask. These flasks were put into the clean, dark, and room-temperature environment for fermentation. The collected samples were filtered through 0.22 μm membranes for subsequent experiments.

### 2.4. Antioxidant Capacities and Total Phenolic Content Assessment

The antioxidant capacities assessment included ferric reducing antioxidant power (FRAP) and Trolox-equivalent antioxidant capacity (TEAC) assays [8,17].

The FRAP assay procedure followed the protocol described in the literature [17] and the results were reported in µmol Fe^2+^/L. The TEAC assay procedure also followed the literature [8] and the results were reported in µmol Trolox/L.

The total phenolic content (TPC) was assessed using the Folin–Ciocalteu method as described in previous literature [8,17], and the results were reported in mg of gallic acid equivalent (GAE)/L.

### 2.5. Evaluation of Bioactive Compounds

The bioactive compounds in kombucha beverages were identified and quantified using high-performance liquid chromatography (HPLC) with a photodiode array detector (PDA) (Waters, Milford, MA, USA), following the previous studies with minor adjustment [8,17]. The Agilent Zorbax Eclipse XDB-C18 column (Santa Clara, CA, USA), with a size of 250 mm × 4.6 mm, 5 µm was used for separation. The gradient elution program was detailed in the literature [8,17].

### 2.6. Sensory Pilot Study

The sensory pilot study of kombucha beverages was conducted based on previous studies [8,17]. Different types of kombucha samples were evaluated by 8 participants (seven graduate students and one professor) from the Department of Nutrition, School of Public Health, Sun Yat-sen University, for their odor, color, flavor, sourness, and overall acceptability. These participants have participated in sensory evaluation of kombucha in our previous studies [8,17] and have rich experience. The hedonic scale ranged from 1–9 levels, where 9 indicated extremely like, 8 indicated greatly like, 7 indicated moderately like, 6 indicated slightly like, 5 indicated neither like nor dislike, 4 indicated slightly dislike, 3 indicated moderately dislike, 2 indicated greatly dislike, and 1 indicated extremely dislike.

### 2.7. Statistical Analysis

All experiments were conducted in triplicate and the results were shown as mean ± standard deviation (SD). Data processing and analysis were performed using Excel 2016 (Microsoft, Washington, DC, USA) and SPSS 25.0 (IBM Corp., Armonk, NY, USA). The one-way analysis of variance (ANOVA) plus post hoc Fisher least significant difference (LSD) was used to test the significance of multiple groups (same kombucha at different fermentation times) and the corresponding pairwise comparisons between groups. The one-way ANOVA was utilized to test the significance of kombucha fermented with residues group and kombucha fermented without residues group. Moreover, the Pearson correlation coefficient was utilized to determine the correlation between parameters and compound concentrations, and heat maps were plotted (https://www.chiplot.online (accessed on 17 May 2023)). In addition, partial least squares regression (PLSR) was also utilized to test the relationships between parameters and compound concentration. The statistical significance was set at *p* < 0.05.

## 3. Results and Discussion

**Figure 1:**
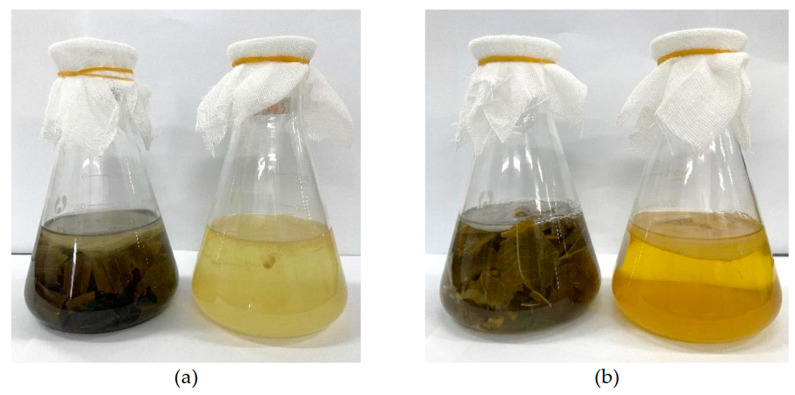
The appearances of kombucha beverages from bamboo leaf and mulberry leaf. (**a**) bamboo leaf kombucha fermented with or without residues; (**b**) mulberry leaf kombucha fermented with or without residues.

In this study, kombucha beverages based on bamboo leaf and mulberry leaf were investigated because both leaves contain many bioactive compounds and possess various bioactivities. The appearances of kombucha beverages fermented by bamboo leaf and mulberry leaf are shown in Figure 1.

### 3.1. Antioxidant Activities

#### 3.1.1. FRAP Values

**Figure 2:**
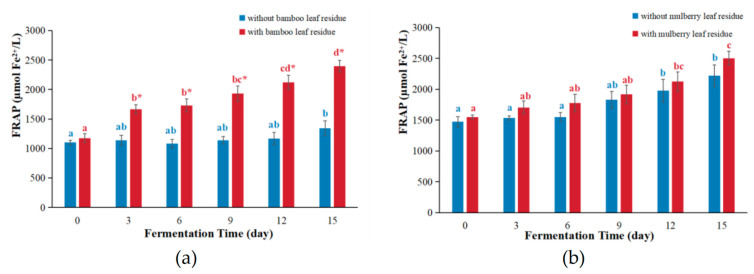
The changes in FRAP values during kombucha fermentation. (**a**) FRAP values of bamboo leaf kombucha; (**b**) FRAP values of mulberry leaf kombucha. In each color, the distinct letter represents significant differences (*p* < 0.05) in kombucha at different fermentation time. The * represents significant difference between kombucha fermented with leaf residues and that without leaf residues under the same fermentation time (*p* < 0.05).

The FRAP assay is commonly used to measure antioxidant capacity in food products by evaluating the reduction ability of substances on ferric ions [17]. The FRAP values of kombucha based on bamboo leaf as well as mulberry leaf are displayed in Figure 2.

For bamboo leaf kombucha fermented with leaf residues, the FRAP values increased over the fermentation period and reached the peak on day 15, which was 2.04 times that of day 0 (Figure 2a). For bamboo leaf kombucha fermented without leaf residues, the FRAP values hardly changed with the prolongation of the fermentation time. Moreover, the FRAP values of bamboo leaf kombucha fermented with leaf residues were remarkedly higher than those of kombucha fermented without leaf residues. This changing tendency was similar to that reported in the literature. For example, a study found that the FRAP value of green tea kombucha fermented with residues was significantly higher than that without residues [17]. Another study also found that fermentation with tea residues enhanced the FRAP values in kombucha based on vine tea and sweet tea compared with those without tea residues [8]. These results might be caused by many reasons. For example, boiling for only 5 min could not extract all the bioactive compounds from plant materials [18] and the rest of the bioactive components could be extracted with the help of enzymes and microbiota during kombucha fermentation. In addition, the FRAP values of bamboo leaf kombucha (both fermented with and without leaf residues) were higher than that of kombucha-fermented soy whey (681.03 μM Fe^2+^/L) [19].

For both mulberry leaf kombucha fermented with and without leaf residues, the FRAP values gradually raised over time and arrived at their peak on day 15, which were 1.62 times and 1.51 times compared with those of day 0, respectively (Figure 2b). Perhaps this was because some bioactive compounds (such as phenolic compounds and flavonoids) could be produced under the action of microorganisms, which could enhance the antioxidant activities of kombucha [20,21]. Moreover, the FRAP values of mulberry leaf kombucha fermented with leaf residues were slightly higher than those of kombucha fermented without leaf residues, although the difference was not statistically significant (*p* > 0.05). The FRAP values of mulberry leaf kombucha (both fermented with and without leaf residues) were higher than that of kombucha-fermented soy whey (681.03 μM Fe^2+^/L) [19].

#### 3.1.2. TEAC Values

**Figure 3:**
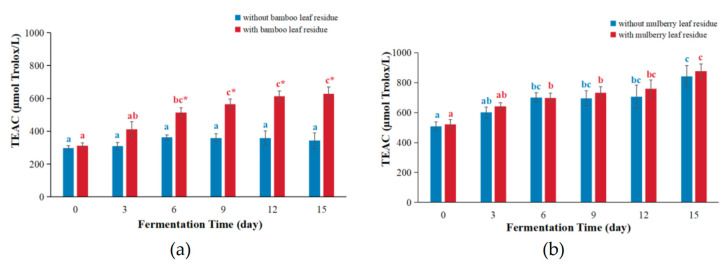
The changes in TEAC values during kombucha fermentation. (**a**) TEAC values of bamboo leaf kombucha; (**b**) TEAC values of mulberry leaf kombucha. In each color, the distinct letter represents significant differences (*p* < 0.05) in kombucha at different fermentation times. The * represents significant difference between kombucha fermented with leaf residues and that without leaf residues under the same fermentation time (*p* < 0.05).

The antioxidant capacity of substances could also be measured using the TEAC assay by comparing their ability to scavenge ABTS^•+^ radical cations with that of a standard reference compound Trolox [8]. The TEAC values of kombucha based on bamboo leaf residues and mulberry leaf residues are shown in Figure 3.

For bamboo leaf kombucha fermented with leaf residues (Figure 3a), the TEAC values raised above fermentation and got to the top on day 15, which was 2.01-fold higher compared with that of day 0. For bamboo leaf kombucha fermented without leaf residues, the TEAC values remained relatively stable and did not change significantly as the fermentation time increased. The TEAC values of bamboo leaf kombucha fermented with leaf residues were significantly higher than those of kombucha fermented without leaf residues, by 1.84-fold on day 15. The reason might also be the same as the FRAP values. In addition, the TEAC values of bamboo leaf kombucha (both with and without leaf residues) were higher than that of black carrot kombucha (53.72 µmol Trolox/L) [22].

The TEAC values of kombucha based on mulberry leaf are shown in Figure 3b. For both mulberry leaf kombucha fermented with and without leaf residues, the TEAC values gradually increased as the fermentation time extended, and reached their maximum on day 15, with 1.68-fold and 1.66-fold increases compared with those of day 0, respectively. Several studies had reported similar results with kombucha prepared with black tea and green tea infusions [23]. Additionally, there was no statistical difference between the mulberry leaf kombucha fermented with leaf residues and that without leaf residues (*p* > 0.05). Moreover, the TEAC values of mulberry leaf kombucha (both fermented with and without residues) were higher than that of black carrot kombucha (53.72 µmol Trolox/L) [22].

For the bamboo leaf kombucha fermented with leaf residue and mulberry leaf kombucha (both fermented with and without leaf residues), the TEAC values were persistently elevated with the prolongation of fermentation time. Another study also obtained a similar tendency to the TEAC values [24]. However, a study found that the TEAC value of kombucha fermented by the green tea infusion was increased before day 7 and then decreased [25]. The differences in the radical scavenging activity observed in various kombucha might be attributed to the difference in the types of fermentation substrates and microbiota present in different kombucha beverages [26].

### 3.2. TPC Values

**Figure 4:**
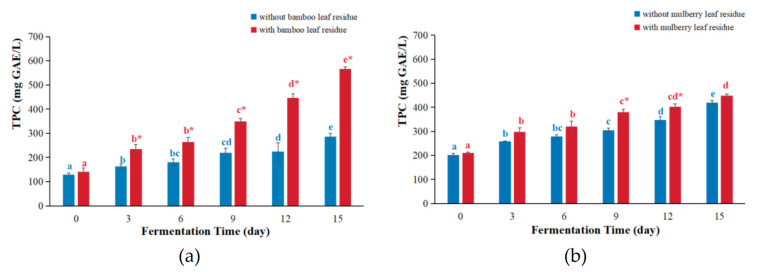
The changes in TPC values during kombucha fermentation. (**a**) TPC values of bamboo leaf kombucha; (**b**) TPC values of mulberry leaf kombucha. In each color, the distinct letter represents significant differences (*p* < 0.05) in kombucha at different fermentation times. The * represents significant difference between kombucha fermented with leaf residues and that without leaf residues under the same fermentation time (*p* < 0.05).

The Folin–Ciocalteu method is a spectrophotometric assay that measures TPC by reacting with phenolic compounds in the sample and is widely used in many studies [17,27]. The TPC values of kombucha based on bamboo leaf and mulberry leaf are shown in Figure 4.

For bamboo leaf kombucha fermented with leaf residues (Figure 4a), the TPC values increased as the fermentation time progressed and reached its maximum on day 15, which was a 4.03-fold increase compared with that of day 0. For bamboo leaf kombucha fermented without leaf residues, the TPC values increased with the extension of the fermentation time and peaked on day 15, which was 2.07 times that of day 0. The tendency was similar to the previous studies [28,29]. Obviously, the TPC values of bamboo leaf kombucha fermented with leaf residues were higher compared with those of kombucha fermented without leaf residues, with a 1.99- and 1.97-fold increase on days 12 and 15. Moreover, the TPC value of bamboo leaf kombucha fermented with leaf residues was higher than those of kombucha fermented with the black tea infusion (412.25 mg GAE/L) and strawberry tree (*Arbutus unedo*) fruits (9.0 mg GAE/100 mL) [30,31].

The TPC values of kombucha based on the mulberry leaf are shown in Figure 4b. For both mulberry leaf kombucha fermented with or without leaf residues, the TPC values gradually raised over time and got to the top on day 15, which were 2.07 times and 2.13 times those of day 0, respectively. Additionally, the TPC values of mulberry leaf kombucha fermented with leaf residues were slightly higher than that of kombucha fermented without leaf residues. Furthermore, the TPC values of mulberry leaf kombucha (both fermented with and without leaf residues) were higher than that fermented with strawberry tree (*Arbutus unedo*) fruits (9.0 mg GAE/100 mL) [31].

In brief, fermentation with leaf residues increased the TPC values in both bamboo leaf and mulberry leaf kombucha. As mentioned before, boiling for only 5 min could not extract all the bioactive compounds from the plant materials [22] and the rest of the polyphenols in the leaves could be extracted over the fermentation period. Moreover, the microbial hydrolysis could increase the degradation of complex polyphenols to small molecules [28,29], which could further increase the TPC values in kombucha fermented with leaf residues.

### 3.3. Concentrations of Bioactive Compounds in Kombucha

**Figure 5:**
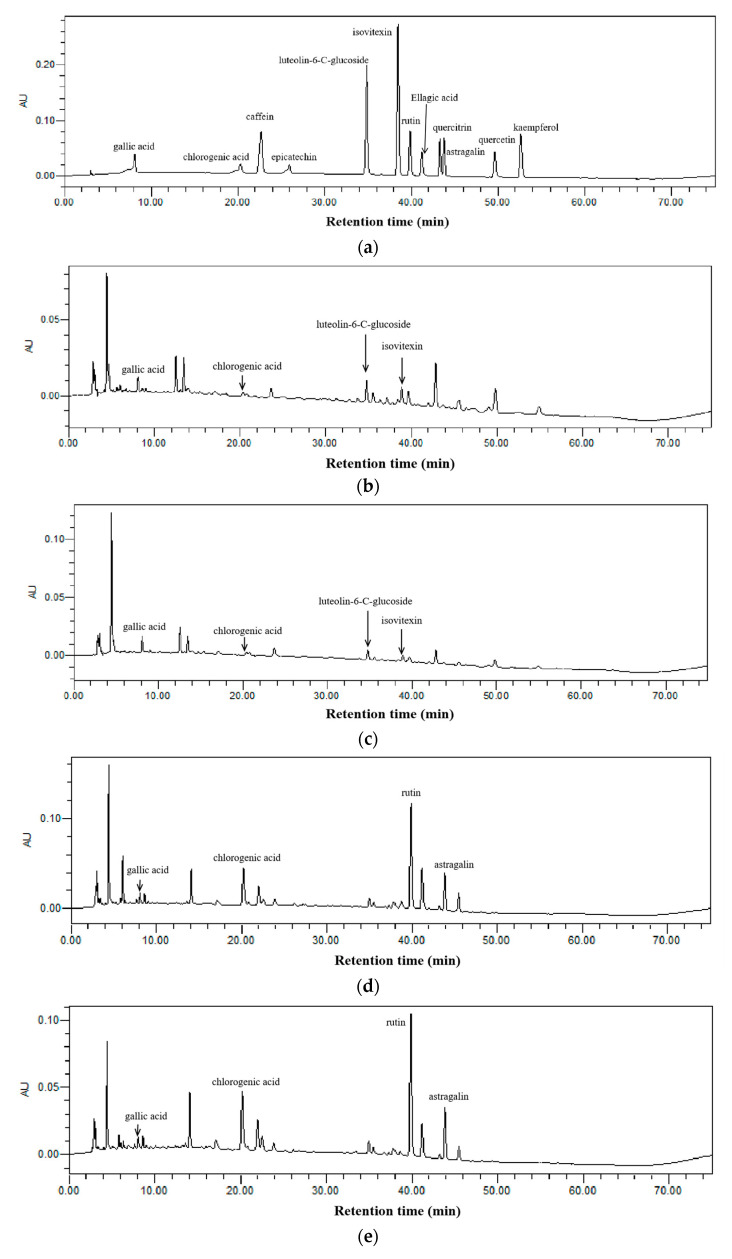
The representative HPLC chromatograms of standards and kombucha beverages at 270 nm. (**a**) standards; (**b**) bamboo leaf kombucha fermented without leaf residues; (**c**) bamboo leaf kombucha fermented with leaf residues; (**d**) mulberry leaf kombucha fermented without leaf residues; (**e**) mulberry leaf kombucha fermented with leaf residues.

The bioactive compounds in kombucha beverages were separated and quantified using HPLC-PDA, as displayed in Figure 5. In bamboo leaf kombucha, four compounds were identified as gallic acid, chlorogenic acid, luteolin-6-C-glucoside, and isovitexin (Figure 5b,c). In mulberry leaf kombucha, four compounds were identified as gallic acid, chlorogenic acid, rutin, and astragalin (Figure 5d,e).

**Figure 6:**
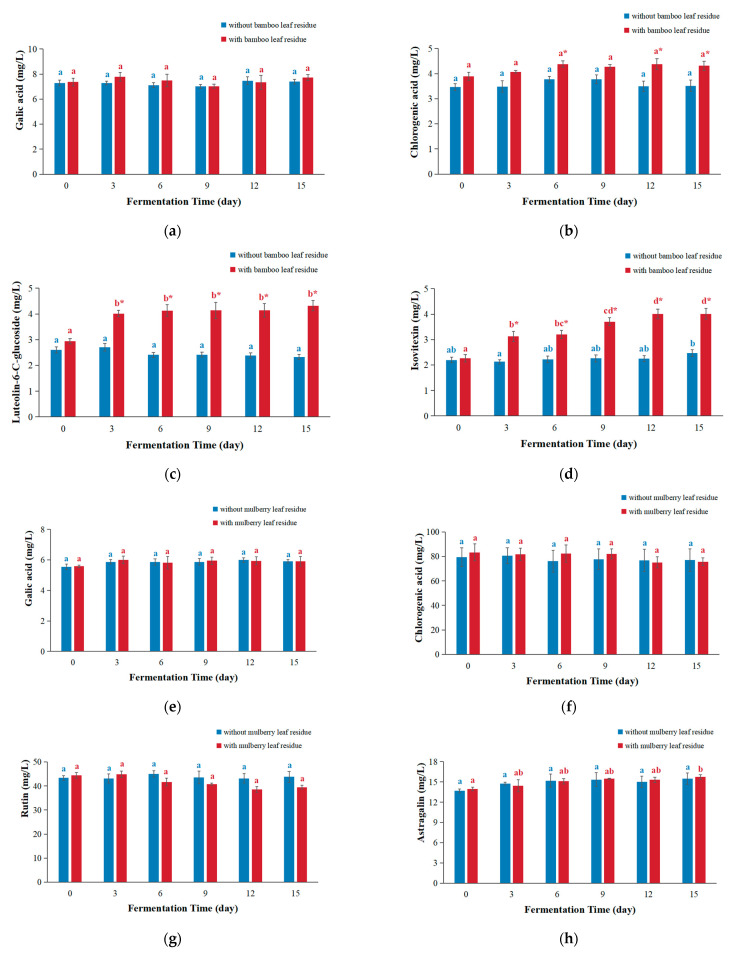
Changes in the contents of main phenolic compounds during kombucha fermentation. (**a**–**d**) bamboo leaf kombucha and (**e**–**h**) mulberry leaf kombucha. In each color, the distinct letter represents significant differences (*p* < 0.05) in kombucha at different fermentation times. The * represents significant difference between kombucha fermented with leaf residues and that without leaf residues under the same fermentation time (*p* < 0.05).

The compounds in kombucha were quantified using the peak area under the maximum absorption wavelength, and the corresponding results are presented in Figure 6.

For bamboo leaf kombucha fermented without leaf residues, the concentrations of gallic acid, chlorogenic acid, luteolin-6-C-glucoside, and isovitexin remained relatively stable and did not change significantly as the fermentation time increased. This might be due to the absence of microorganisms or enzymes in kombucha that can degrade these compounds. For bamboo leaf kombucha fermented with leaf residues, there was no significant change observed in the concentrations of gallic acid and chlorogenic acid with the progression of the fermentation time (Figure 6a,b), which showed differences from kombucha based on black tea and green tea [8,17]. A study found that the content of gallic acid significantly increased through the enzymatic degradation of green tea extract with tannase [32]. Therefore, it could be due to the absence of compounds that could be degraded to produce gallic acid and chlorogenic acid in bamboo leaf residues. The concentrations of luteolin-6-C-glucoside increased on day 3 and then stayed stable ((Figure 6c), which might be because luteolin-6-C-glucoside continued to dissolve from leaf residues during the first three days and almost completely dissolved at day 3. The concentrations of isovitexin were increased over the prolonged fermentation period and reached the peak on day 12 (Figure 6d), which might be because the isovitexin kept dissolving from leaf residues until it was completely dissolved. Compared with the bamboo leaf kombucha without leaf residues, the concentrations of chlorogenic acid were higher in the bamboo leaf kombucha fermented with leaf residues, with 1.16, 1.25, and 1.23 times on days 6, 12, and 15, respectively; the concentrations of luteolin-6-C-glucoside and isovitexin were higher in the kombucha fermented with bamboo leaf residues, being 1.78 and 1.86 times higher on days 12 and 15, respectively. Additionally, the concentration of gallic acid in bamboo leaf kombucha fermented with leaf residues was similar to those of kombucha fermented without leaf residues. Generally speaking, these results suggested that fermentation with leaf residues could be helpful for the full extraction of bioactive compounds in bamboo leaf.

For mulberry leaf kombucha fermented without leaf residues, the concentrations of gallic acid, chlorogenic acid, rutin, and astragalin did not show significant changes with the prolongation of the fermentation time (Figure 6e–h). For mulberry leaf kombucha fermented with leaf residues, the concentration of gallic acid, chlorogenic acid, and rutin did not change with the prolongation of the fermentation time. Moreover, the concentration of astragalin slightly raised over time and got the highest level on day 15. In addition, there was no statistically significant difference in the concentrations of these compounds between the mulberry leaf kombucha fermented with leaf residues and that without leaf residues.

Furthermore, although the FRAP, TEAC, and TPC values of the bamboo leaf or mulberry leaf kombucha beverages were lower than those of black tea or green tea kombucha beverages [17], the different components in these beverages could result in varying health benefits, and the consumers could choose different beverages according to their demand. Especially because of the absence of caffeine in bamboo leaf and mulberry leaf kombucha beverages, for individuals sensitive to caffeine, bamboo leaf or mulberry leaf kombucha beverages could be better choices than black tea and green tea kombucha beverages, which contain high concentrations of caffeine.

### 3.4. Correlations between Parameters and Concentrations of Compounds

**Figure 7:**
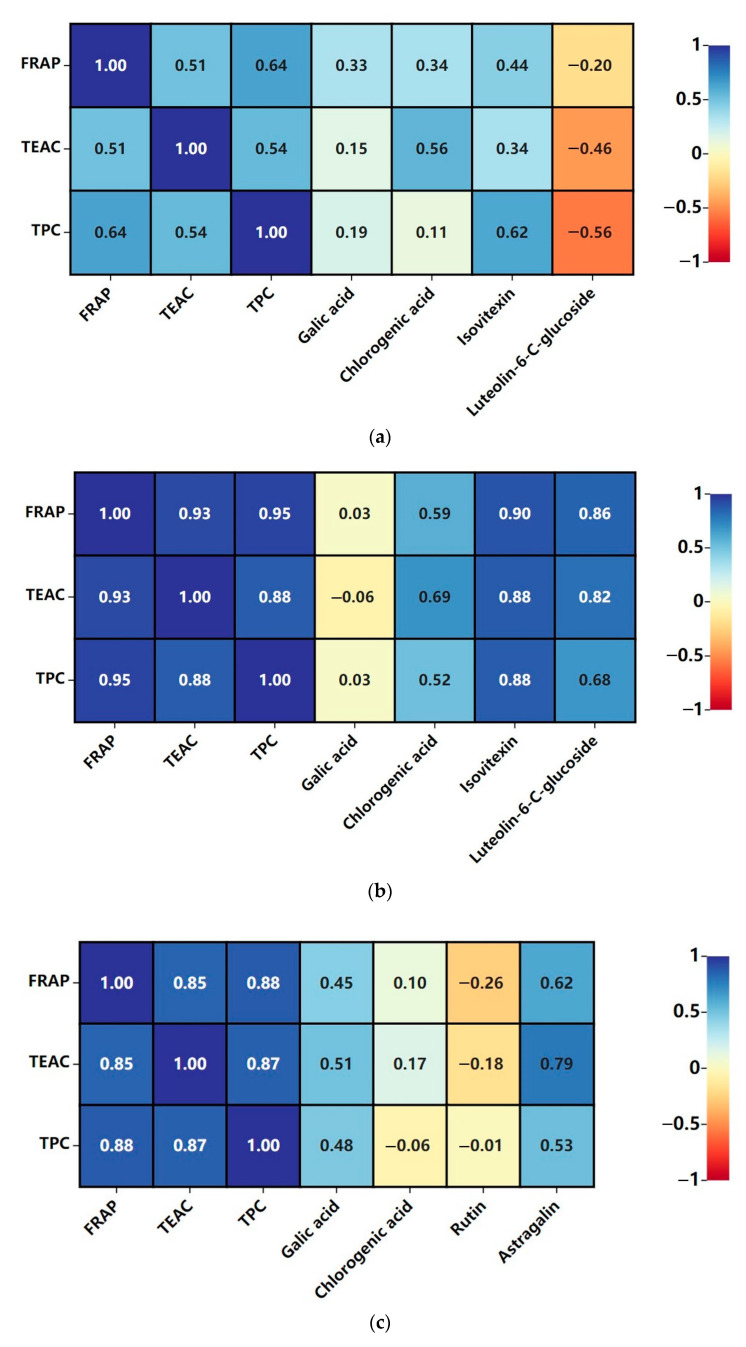

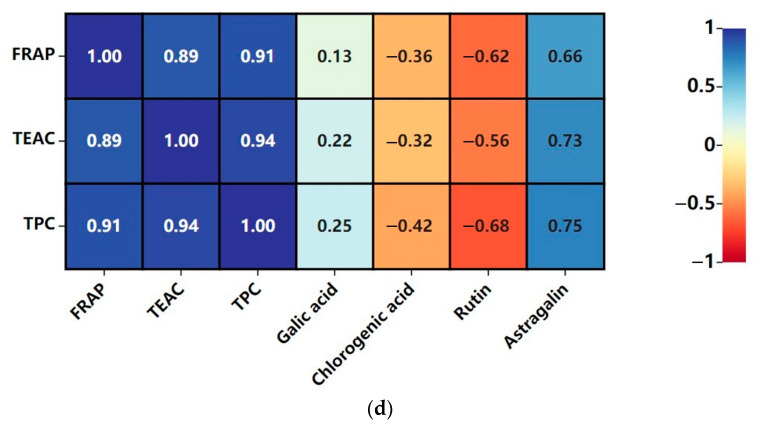
Heat map analysis of compound concentrations and associated parameters. (**a**) bamboo leaf kombucha fermented without leaf residues; (**b**) bamboo leaf kombucha fermented with leaf residues; (**c**) mulberry leaf kombucha fermented without leaf residues; (**d**) mulberry leaf kombucha fermented with leaf residues.

The correlations between FRAP, TEAC, TPC, and concentrations of compounds are obtained through heat map visualization and are shown in Figure 7.

For FRAP values vs. TEAC values, significant correlations were found in bamboo leaf kombucha fermented with leaf residues (R = 0.93), mulberry leaf kombucha fermented without leaf residues (R = 0.85), and mulberry leaf kombucha fermented with leaf residues (R = 0.89), which suggested that the antioxidant compounds in these beverages not only had the ability to reduce ferric ions, but also scavenge ABTS^•+^ radical cations. For FRAP values vs. TPC values, they were highly correlated in bamboo leaf kombucha without (R = 0.64) or with (R = 0.95) leaf residues, and mulberry leaf kombucha without (R = 0.88) or with (R = 0.91) leaf residues. The findings suggested that the phenolic compounds could be contributed to the ability to reduce Fe^3+^. For TEAC values vs. TPC values, there were significant correlations in bamboo leaf kombucha fermented with leaf residues (R = 0.88), mulberry leaf kombucha fermented without leaf residues (R = 0.87), and mulberry leaf kombucha fermented with leaf residues (R = 0.94), which indicated that phenolic compounds might be major contributors to the free radical scavenging function of kombucha beverages.

With regards to the correlation between FRAP values and bioactive compounds in bamboo leaf kombucha, significant correlations were found between the FRAP values and concentration of isovitexin (R = 0.90), as well as the FRAP values and the concentration of luteolin-6-C-glucoside (R = 0.86) in bamboo leaf kombucha fermented with leaf residues, suggesting that the isovitexin and luteolin-6-C-glucoside could potentially contribute to the antioxidant capacity. Moreover, the studies reported that isovitexin and luteolin were major flavonoid carbon glycosides in bamboo leaf that contributed to its antioxidant activity [33,34,35], which aligned with the outcomes obtained in this study. For the correlation between the FRAP values and bioactive compounds in mulberry leaf kombucha, significant correlations were found between the FRAP values and the concentration of astragalin in mulberry leaf kombucha fermented without leaf residues (R = 0.62), as well as the FRAP values and the concentration of astragalin in mulberry leaf kombucha fermented with leaf residues (R = 0.66).

With regards to the correlation between TEAC values and bioactive compounds in bamboo leaf kombucha, significant correlations were found between TEAC values and several compounds, including chlorogenic acid (R = 0.69), isovitexin (R = 0.88), and luteolin-6-C-glucoside (R = 0.82), in bamboo leaf kombucha fermented with leaf residues. For the correlation between the TEAC values and bioactive compounds in mulberry leaf kombucha, significant correlations existed between the TEAC values and astragalin (R = 0.79) in mulberry leaf kombucha fermented without leaf residues, and between the TEAC values and rutin (R = 0.73) in mulberry leaf kombucha fermented with leaf residues.

With regards to the correlation between TPC values and bioactive compounds in bamboo leaf kombucha, significant correlations were found between the TPC values and several compounds, including isovitexin (R = 0.88) and luteolin-6-C-glucoside (R = 0.68), in bamboo leaf kombucha fermented with leaf residues. Moreover, TPC values were related to isovitexin (R = 0.62) in bamboo leaf kombucha fermented without leaf residues. For the correlation between the TPC values and bioactive compounds in mulberry leaf kombucha, the TPC values were related to astragalin (R = 0.75) in mulberry leaf kombucha fermented with leaf residues.

**Figure 8:**
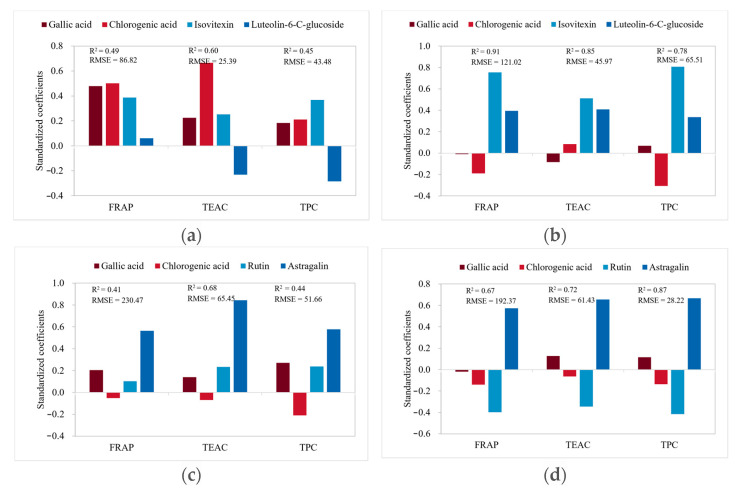
Standardized coefficients in PLSR models. (**a**) bamboo leaf kombucha fermented without leaf residues; (**b**) bamboo leaf kombucha fermented with leaf residues; (**c**) mulberry leaf kombucha fermented without leaf residues; (**d**) mulberry leaf kombucha fermented with leaf residues. RMSE, root mean square error.

**Figure 9:**
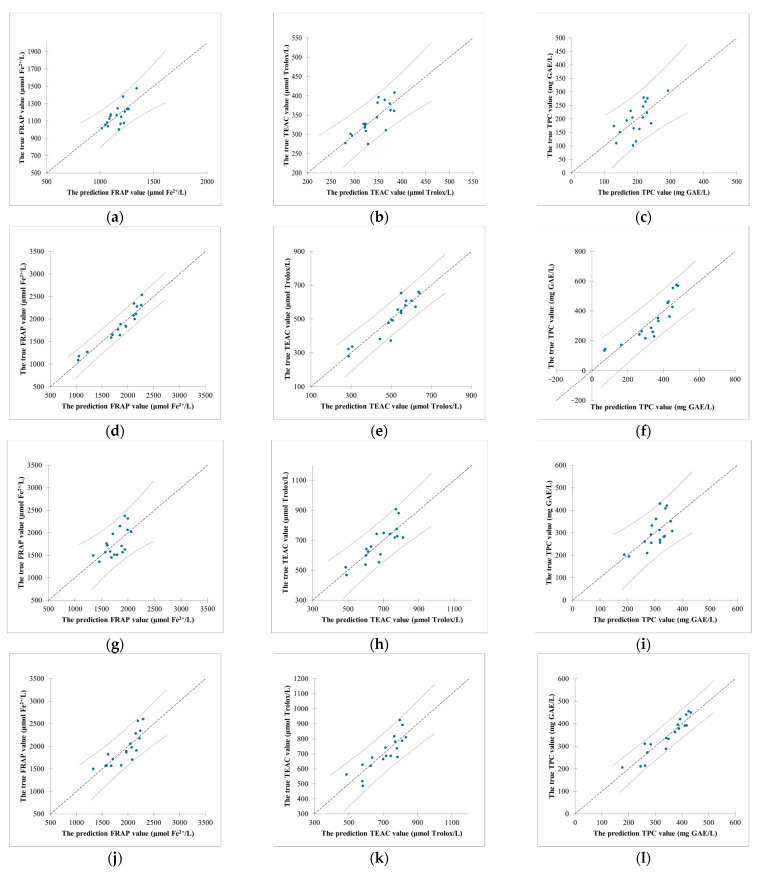
The correlation between the predicted values and true values. (**a**–**c**) bamboo leaf kombucha fermented without leaf residues; (**d**–**f**) bamboo leaf kombucha fermented with leaf residues; (**g**–**i**) mulberry leaf kombucha fermented without leaf residues; (**j**–**l**) mulberry leaf kombucha fermented with leaf residues.

On the other hand, the correlations between the concentrations of compounds and the parameters were also studied using the PLSR model and the optimum number of PLS-factors required for the models was three. The results are displayed in Figure 8 and Figure 9.

For bamboo leaf kombucha fermented without leaf residues (Figure 8a and Figure 9a–c), gallic acid, chlorogenic acid, and isovitexin generally had positive impacts on the FRAP, TEAC, and TPC values. Moreover, the prediction errors were small in the FRAP, TEAC, and TPC values, suggesting high prediction accuracy. For bamboo leaf kombucha fermented with leaf residues (Figure 8b and Figure 9d–f), isovitexin and luteolin-6-C-glucoside had positive impacts on the FRAP, TEAC, and TPC values, which were consisted with those of the Pearson correlation coefficients. Moreover, the R^2^ were high (0.91, 0.85, and 0.78, respectively), as well as the prediction errors being small in the FRAP, TEAC, and TPC values. The results showed that the model performed well. For mulberry leaf kombucha (both fermented without and with leaf residues), astragalin had strong positive impacts on the FRAP, TEAC, and TPC values, which were similar to those of the Pearson correlation coefficients. In addition, the R^2^ were generally high in these models, which indicated models performed well. Furthermore, the prediction errors were small in the TEAC and TPC values, suggesting high prediction accuracy (Figure 8c,d and Figure 9g–l).

Although the results for the correlations between the concentrations of compounds and parameters from the Pearson correlation coefficients and PLSR model had little differences, they were generally similar.

### 3.5. Sensory Pilot Study

**Figure 10:**
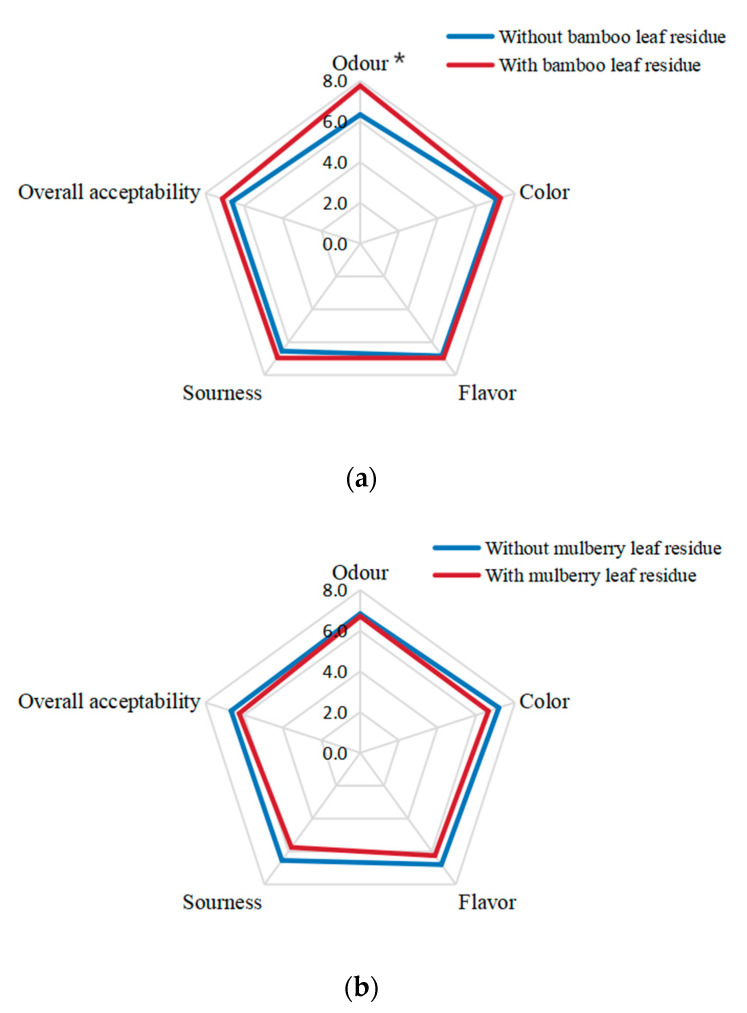
Sensory pilot study of kombucha beverages. (**a**) bamboo leaf kombucha fermented with or without leaf residues; (**b**) mulberry leaf kombucha fermented with or without leaf residues. The * represents significant difference between kombucha fermented with leaf residues and that without leaf residues (*p* < 0.05).

The sensory pilot study included odor, color, flavor, sourness, and overall acceptability of the kombucha beverages. The findings are displayed in Figure 10.

For bamboo leaf kombucha, the odor of kombucha fermented with bamboo leaf residues was better than those without leaf residues (*p* < 0.05). The scores of sourness and overall acceptability of kombucha fermented with bamboo leaf residues were slightly higher than those without leaf residues, although the differences showed not statistically significant (*p* > 0.05). Moreover, fermentation with or without residues had no difference on the color and flavor of the bamboo leaf kombucha.

For mulberry leaf kombucha, the color, flavor, sourness, and overall acceptability of the kombucha fermented without bamboo leaf residues were slightly better than those with leaf residues, but the differences were not statistically significant (*p* > 0.05). Moreover, fermentation with or without residues had no difference on the odor of the mulberry leaf kombucha.

In general, fermentation with leaf residues could enhance the hedonic scores of bamboo leaf kombucha to some extent, while it could not enhance the sensory acceptability of mulberry leaf kombucha. Moreover, among the four kombucha beverages in this study, bamboo leaf kombucha fermented with leaf residues got the highest scores in each indicator.

## 4. Conclusions

In this study, mulberry leaf kombucha and bamboo leaf kombucha have been explored. The results suggested that fermentation with leaf residues could markedly increase the FRAP, TEAC, and TPC values in bamboo leaf kombucha beverages with an enhancement of the sensory acceptability, but the enhancement in mulberry leaf kombucha beverages was not statistically significant, except for the TPC values. Moreover, several bioactive compounds in these kombucha beverages were separated and quantified using HPLC-PDA, and they might contribute to the antioxidant capacities of these beverages. Fermentation with leaf residues increased the concentrations of certain bioactive compounds in bamboo leaf kombucha beverages, but not in mulberry leaf kombucha beverages. Overall, fermentation with leaf residues might be a more appropriate option for making bamboo leaf kombucha beverages, while both fermentation with and without leaf residues showed no difference for making mulberry leaf kombucha beverages. In total, this study found that bamboo leaf and mulberry leaf could be used as alternative substrates to produce kombucha beverages with strong antioxidant activities and various bioactive compounds, which could be used to prevent and manage certain oxidative-stress-related diseases. In addition, this study could be helpful for the value-added utilization of mulberry and bamboo leaves.

## Data Availability

Data are contained within the article.
